# Significant Correlation of Dynamic Pan-Immune Inflammation Value and Magnetic Resonance Imaging Response in Patients with Locally Advanced Rectal Cancer Treated with Total Neoadjuvant Therapy

**DOI:** 10.3390/curroncol33050278

**Published:** 2026-05-09

**Authors:** Sukran Celikarslan, Duygu Karahacioglu, Bengi Gurses, Fatih Selcukbiricik, Emre Balik, Derya Salim Uymaz, Ahmet Rencuzogullari, Dursun Bugra, Sahin Lacin, Kerim Kaban, Perran Fulden Yumuk, Nil Molinas Mandel, Ayse Armutlu, Burcu Saka, N. Volkan Adsay, Metin Vural, Merve Duman, Caglayan Selenge Beduk Esen, Nulifer Kilic Durankus, Duygu Sezen, Saliha Ezgi Oymak, Yasemin Atagun, Ugur Selek

**Affiliations:** 1 Department of Radiation Oncology, Koç University, Istanbul 34450, Türkiye; scelikarslan@kuh.ku.edu.tr (S.C.); ndurankus@kuh.ku.edu.tr (N.K.D.); dsezen@kuh.ku.edu.tr (D.S.); ybolukbasi@kuh.ku.edu.tr (Y.A.); 2 Department of Radiology, Koç University, Istanbul 34450, Türkiye; dkarahacioglu@kuh.ku.edu.tr (D.K.); bgurses@kuh.ku.edu.tr (B.G.); 3 Department of Medical Oncology, Koç University, Istanbul 34450, Türkiye; fselcukbiricik@kuh.ku.edu.tr (F.S.); salacin@kuh.ku.edu.tr (S.L.); fyumuk@kuh.ku.edu.tr (P.F.Y.); nmandel@kuh.ku.edu.tr (N.M.M.); 4 Department of General Surgery, Koç University, Istanbul 34450, Türkiye; ebalik@ku.edu.tr (E.B.); duymaz@kuh.ku.edu.tr (D.S.U.); arencuzogullari@kuh.ku.edu.tr (A.R.); dbugra@ku.edu.tr (D.B.); 5 Department of Medical Oncology, Vehbi Koç Foundation American Hospital, Istanbul 34365, Türkiye; kerimk@amerikanhastanesi.org; 6 Department of Pathology, Koç University, Istanbul 34450, Türkiye; aarmutlu@kuh.ku.edu.tr (A.A.); bsaka@kuh.ku.edu.tr (B.S.); vadsay@kuh.ku.edu.tr (N.V.A.); 7 Department of Radiology, VKF American Hospital, Istanbul 34365, Türkiye; metinv@amerikanhastanesi.org; 8 Department of Radiation Oncology, Bağcılar Training and City Hospital, Istanbul 34200, Türkiye; caglayanmerve.itf@gmail.com; 9 Department of Radiation Oncology, VKF American Hospital, Istanbul 34365, Türkiye; caglayane@amerikanhastanesi.org (C.S.B.E.); eoymak@amerikanhastanesi.org (S.E.O.)

**Keywords:** locally advanced rectal cancer, total neoadjuvant therapy, magnetic resonance imaging, tumor regression grade, pan-immune inflammation value, non-operative management, dynamic biomarker, regrowth

## Abstract

**Background**: Accurate identification of complete or near-complete responders after total neoadjuvant therapy (TNT) is critical for selecting candidates for non-operative management in locally advanced rectal cancer (LARC). While static inflammatory biomarkers have been widely investigated, the predictive value of dynamic changes in systemic inflammation during TNT remains unclear. This study evaluated whether changes in the Pan-Immune Inflammation Value (PIV) correlate with magnetic resonance imaging tumor regression grade (mrTRG) and regrowth risk. **Methods**: Seventy-three patients with LARC treated with TNT between 2018 and 2024 were retrospectively analyzed. Patients were classified as complete/near-complete responders (C-NCRG; mrTRG1–2) or residual disease group (RDG; mrTRG3–5). The PIV was calculated as (platelet × monocyte × neutrophil)/lymphocyte at treatment initiation (first-PIV), three weeks after completion of chemotherapy (last-PIV), and at regrowth (Rg-PIV). Dynamic changes were defined as Δ-PIV (last-PIV minus first-PIV) and ΔRg-PIV (Rg-PIV minus first-PIV). Receiver operating characteristic analysis identified optimal cutoffs, and logistic regression assessed independent associations. **Results**: The baseline PIV did not differ between groups. Last-PIV and Δ-PIV were significantly lower in the C-NCRG compared with RDG (*p* = 0.006). A Δ-PIV cutoff of −36.9 discriminated responders (AUC = 0.705; sensitivity 53.7%; specificity 84.4%) and remained independently associated with MRI response in multivariate analysis. In the non-operative management cohort, ΔRg-PIV was significantly lower in patients without regrowth (*p* = 0.001), with a cutoff of −77.72 (AUC = 0.794; sensitivity 88.9%; specificity 66.7%). Overall survival was superior in the C-NCRG (*p* = 0.01). **Conclusions**: A dynamic reduction in the PIV during TNT is significantly associated with favorable MRI response and lower regrowth risk in LARC. PIV dynamics may serve as a noninvasive complementary biomarker to support response assessment and stratification in organ-preservation strategies.

## Simple Summary

Patients with locally advanced rectal cancer often receive intensive preoperative treatment known as total neoadjuvant therapy, which can allow some patients to avoid surgery if a complete response is achieved. Determining who can safely undergo non-operative management remains challenging. Blood-based inflammatory markers have shown promise as predictors of treatment response, but most studies have evaluated only baseline values. In this study, we examined whether changes in the Pan-Immune Inflammation Value—a marker calculated from routine blood counts—are associated with magnetic resonance imaging (MRI) response and tumor regrowth risk. We found that a reduction in this marker during treatment was linked to better MRI response and a lower likelihood of regrowth. These findings suggest that dynamic changes in systemic inflammation may provide a simple, accessible tool to complement imaging in guiding personalized treatment strategies.

## 1. Introduction

Although curative, total mesorectal excision (TME) is associated with significant morbidity, including sexual dysfunction, urinary dysfunction, and the potential for a permanent stoma, all of which profoundly impact a patient’s quality of life [1]. Total neoadjuvant therapy (TNT) protocols have been a game-changer, providing high complete response rates and offering non-operative management (NOM) alternatives to TME in the treatment of locally advanced rectal cancer (LARC). In the RAPIDO, PRODIGE-23, and STELLAR phase III trials, total neoadjuvant therapy (TNT) significantly improved pathological and clinical complete response (pCR and cCR) rates compared to standard chemoradiotherapy (CRT), and TNT also led to superior disease-free survival (DFS) across all these studies [2,3,4]. These findings are further supported by systematic reviews and meta-analyses confirming higher pCR rates with TNT [5,6]. The OPRA trial, which demonstrated that TNT enables organ preservation in approximately half of patients through NOM without compromising survival, underscores the clinical imperative for accurate, noninvasive, and accessible methods for identifying NOM candidates [7]. A recent pooled analysis of the CAO/ARO/AIO-12 and OPRA multicenter, randomized phase 2 trials documented that disease-free survival (DFS) was similar in both TNT sequences of chemoradiotherapy (CRT) plus induction or consolidation chemotherapy (CT), while highlighting higher rates of CR for NOM with CRT and consolidation CT [8].

Determining cCR after TNT is a critical step in guiding subsequent management decisions, as the most effective and recommended methods for identifying candidates for NOM include digital rectal examination, endoscopy, and MRI restaging [9]. In the MERCURY prospective cohort trial—a foundational study—MRI-based tumor regression grade (mrTRG) was shown to significantly correlate with both overall survival (OS) and DFS, when compared to pathological staging [10]. There is no doubt about the prognostic value of MRI-based tumor regression grading because of its ability to effectively differentiate between poor and good responders. The prognostic and predictive significance of MRI response in rectal cancer has also been validated by other studies using the MERCURY grading system [11,12]. Locally advanced rectal cancer is typically defined as tumors extending beyond the muscularis propria (cT3–4) and/or with regional lymph node involvement (cN+), as determined by MRI-based staging.

In addition to witnessing the solid response via digital rectal examination, endoscopy, and MRI, systemic inflammation markers are another group of blood parameters that have been extensively investigated in the literature, showing a correlation with both pathological response and prognosis, such as the Pan-Immune-Inflammation Value (PIV) established in patients with colorectal cancer, integrating key indicators of systemic inflammation with neutrophils, lymphocytes, monocytes, and platelets [13]. Preoperative lower PIVs were indicated to predict higher pCR, as well as increased OS and progression-free survival (PFS) [14], while the Italian multi-institutional PILLAR trial demonstrated a strong correlation between inflammation parameters and pCR in LARC patients treated with neoadjuvant CRT [15]. A broader landscape of more tumor-specific biomarkers, such as circulating tumor DNA (ctDNA, radiomics, and AI-based imaging) are emerging [16]. Despite the emergence of these other tools, the PIV remains a valuable tool due to its widespread accessibility, cost-effectiveness and its ability to reflect the host’s systemic immune response, which is distinct from tumor-specific DNA [17]. While static inflammatory markers have been extensively investigated, the exploration into dynamic changes in these markers, particularly within the context of TNT, remains largely underexplored. While static inflammatory biomarkers have been widely studied, they provide only a single timepoint assessment and may not fully capture treatment-related biological dynamics. In contrast, dynamic changes in systemic inflammation may better reflect real-time tumor–host interactions and complement established imaging and emerging molecular biomarkers. This study aims to explore that dynamic changes in the PIV may serve as a predictive parameter for MRI-based tumor regression grade (mrTRG) in LARC following TNT.

## 2. Materials and Methods

### 2.1. Participants

**Figure 1:**
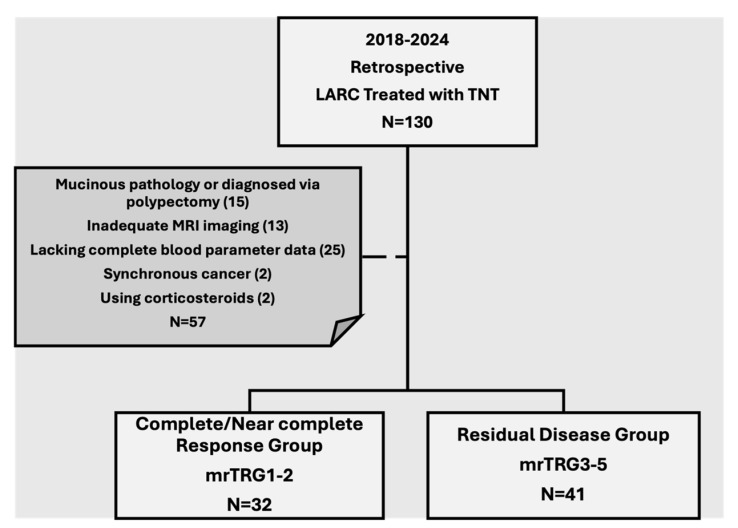
Flowchart of patient inclusion and exclusion criteria.

Between 2018 and 2024, patients diagnosed with LARC who underwent TNT were evaluated. Patients were included if they had undergone rectal staging MRI both at diagnosis and at the end of treatment and had available blood parameters at the initiation of treatment and three weeks after the last chemotherapy cycle. The reason for using blood parameters obtained at least 3 weeks after the last chemotherapy cycle is that the effect of chemotherapy has subsided. Furthermore, these patients did not undergo any adjunctive therapy aimed at elevating platelet or leukocyte levels. Patients with mucinous adenocarcinoma or those diagnosed via polypectomy were excluded due to the inherent limitations in accurately assessing tumor response using MRI. To avoid potential biases in the evaluation of inflammatory markers, patients with pre-existing hematologic or immunosuppressive disorders and those using corticosteroids were also excluded from the study cohort (Figure 1).

### 2.2. Total Neoadjuvant Treatment Protocol

The institutional TNT protocol is a sandwich model including induction chemotherapy (3 cycles of CAPOX: oxaliplatin 130 mg/m^2^ on day 1 plus capecitabine 1650 mg/m^2^ twice daily on days 1–14, every 3 weeks; or 4 cycles of FOLFOX: oxaliplatin 85 mg/m^2^, leucovorin 400 mg/m^2^, 5–FU 400 mg/m^2^ bolus followed by 2400 mg/m^2^ continuous infusion over 46 h, every 2 weeks), CRT (50 Gy in 25 fractions of 5 fractions/week with concomitant capecitabine 1650 mg/m^2^ twice daily on radiotherapy days or infusional 5–FU 225 mg/m^2^/day), and consolidation chemotherapy (3 cycles of CAPOX or 4 cycles of FOLFOX), while allowing an alternative schedule of CRT and consolidation chemotherapy as FOLFOX (4–6 cycles) or CAPOX (3–6 cycles).

### 2.3. Magnetic Resonance Imaging Protocol

All patients underwent MRI using a dedicated rectal cancer protocol to ensure comprehensive locoregional staging. Tumor location, T and N stages (including mesorectal and pelvic lymph nodes), extramural venous invasion (EMVI), and tumor deposits were recorded both at the time of diagnosis and eight–twelve weeks after completion of chemoradiotherapy to assess the treatment response. Tumor response was evaluated using identical criteria (modified from classical mrTRG) to the MERCURY study group (Table 1) [18].

This grading differs slightly from classical mrTRG, incorporating diffusion-weighted imaging in addition to the T2-weighted sequence [19]. According to this grading system, mrTRG grade 1 indicates complete response, mrTRG2 indicates near-complete response, and from mrTRG3 to mrTRG5 there is an increased load of residual tumor [10]. Based on their mrTRG scores, patients were categorized into two groups: the complete/near-complete response group (C-NCRG) (TRG 1–2) and the residual disease group (RDG) (TRG 3–5).

### 2.4. Non-Operative Management Criteria

Eligibility for non-operative management is restricted to patients who achieve either a complete or a near-complete clinical response, as determined by a multidisciplinary tumor board.

Clinical complete response was defined according to standardized criteria and requires fulfillment of all of the following [20]: (1) absence of residual tumor or smooth, flat lesion on digital rectal examination, (2) absence of residual mucosal abnormality on endoscopy other than a flat scar, whitening of the mucosa, or telangiectasia, and (3) fibrotic, linear scar with low signal intensity on T2-weighted images, without residual diffusion restriction or suspicious lymph nodes on MRI.

A near-complete clinical response is considered when the following features are observed [21]: (1) smooth induration or superficial minor mucosal irregularity on digital rectal examination, (2) endoscopic residual findings limited to small irregular mucosal nodules, superficial ulceration, or mild persistent erythema, (3) T2-weighted MRI with downstaging with or without residual fibrosis, small area of residual signal, and complete or partial regression of lymph nodes, and (4) persistence of a small, focal area of high signal intensity on DWI.

Although NOM eligibility is primarily based on complete or near-complete clinical response, final decisions were made by a multidisciplinary tumor board. Therefore, a small subset of patients with mrTRG3 but favorable endoscopic findings were also included in the NOM cohort.

### 2.5. Pan-Immune Inflammation Value Parameters

The Pan-Immune Inflammation Value (PIV) was calculated as (platelet × monocyte × neutrophil)/lymphocyte. The PIV was recorded at the initiation of chemotherapy (first-PIV) and three weeks after completion of the final chemotherapy cycle (last-PIV). To evaluate treatment-related changes, Δ-PIV was defined as the signed difference between last-PIV and first-PIV (Δ-PIV = last-PIV − first-PIV). Accordingly, negative Δ-PIVs indicate a decrease in systemic inflammatory burden following total neoadjuvant therapy (TNT), whereas positive values reflect an increase. For patients managed with the non-operative management (NOM) protocol, an Rg-PIV was calculated using blood parameters at the time of regrowth; in patients without regrowth, values obtained at the median time to regrowth of the cohort were used. This approach was used to approximate a comparable timepoint; however, it may introduce bias and should be interpreted with caution. The ΔRg-PIV was similarly defined as the signed difference between Rg-PIV and first-PIV.

### 2.6. Statistics

The primary objective of this study was to analyze the potential correlation between the PIV and mrTRG in patients diagnosed with LARC. Continuous variables were presented as the mean ± standard deviation (SD), while categorical variables were expressed as frequencies and percentages. Data with a normal distribution were compared using Student’s t-test, whereas the Mann–Whitney U test was employed for non-normally distributed data. The most optimal PIV cutoff was determined using a receiver operating characteristic (ROC) curve analysis to determine the relationship between C-NCRG and RDG. In addition, ROC analysis was performed to determine the PIV cutoff in the NOM patient group between those with and without regrowth.

The potential impact of various risk factors on the C-NCRG and RDG were analyzed using binary logistic regression. Only variables with a *p*-value < 0.2 in univariate analysis were included in the multivariable model.

Also, univariate and multivariate logistic regression analysis was performed in the NOM group to assess the potential factors for regrowth. The initial MRI T stage was dichotomized into early (T3ab) and advanced stages (T3cd–4ab) for the statistical analysis; additionally, due to the small number of patients with proximal tumor localization, these patients were included in the analysis within the group of patients with middle tumor localization. Also, overall survival (OS) was estimated using the Kaplan–Meier method. All statistical analyses were performed using two-sided tests with a significance level of <0.05.

## 3. Results

### 3.1. Patients’ Baseline and Treatment Characteristics

Of the 130 patients, 73 who met the inclusion criteria were retrospectively analyzed. The median follow-up period was 31 months (range: 6–72 months). Tumor location was distal in 35.6% of cases, middle in 61.6%, and proximal in 2.7% of cases. In the vast majority of patients (83.6%), the TNT protocol was the sandwich protocol, while CRT followed by consolidation CT was used in the rest. Table 2 summarizes the distribution of baseline MRI findings across the entire patient population and within the individual response groups.

### 3.2. Pan-Immune Inflammation Values

**Figure 2:**
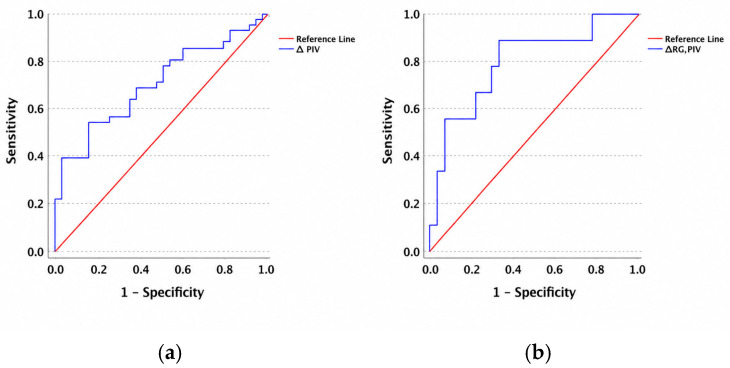
(**a**) Δ-PIV and response group correlation; (**b**) ΔRg-PIV and regrowth status correlation.

Baseline first-PIVs were comparable between the C-NCRG and RDG (mean ± SD: 421.8 ± 207.6 vs. 397.2 ± 271.9; *p* = 0.38). In contrast, last-PIVs were significantly lower in the C-NCRG compared with the RDG (224.5 ± 132.3 vs. 444.8 ± 420.6; *p* = 0.006). Consistent with this finding, the Δ-PIV differed significantly between groups (−197.2 ± 210.7 vs. 47.49 ± 402.5; *p* = 0.006), indicating a marked post-treatment reduction in systemic inflammation among patients achieving complete/near-complete MRI response. The ROC analysis identified a Δ-PIV threshold of −36.9 for discriminating C-NCRG from RDG (AUC = 0.705; sensitivity 53.7%; specificity 84.4%) (Figure 2a).

Within the NOM cohort, ΔRg-PIV was significantly lower in patients without regrowth compared to those with regrowth (−103.23 ± 197.79 vs. 169.71 ± 327.76; *p* = 0.001). ROC analysis yielded a cutoff value of −77.72 (AUC = 0.794; sensitivity 88.9%; specificity 66.7%) (Figure 2b) for distinguishing regrowth status.

### 3.3. Univariate and Multivariate Analysis

In the entire cohort for the prediction of the C-NCRG; univariate analyses were initially performed for the following variables: tumor location, initial T stage, mesorectal lymph node, pelvic lymph node, EMVI, tumor deposit, and Δ-PIV. Among these variables, T stage, mesorectal lymph node, pelvic lymph node, EMVI, tumor deposit, and Δ-PIV were included in the multivariate analysis. It was determined that the significance of the Δ-PIV persisted in the multivariate analysis. The detailed results of these analyses are presented in Table 2.

In the NOM group, potential predictors of tumor regrowth were first assessed using univariate analyses for tumor location, T stage, mesorectal lymph node, pelvic lymph node, EMVI, tumor deposit, and ΔRg-PIV. Variables found to be relevant—tumor location, T stage, and ΔRg-PIV—were subsequently included in the multivariate analysis. Both T stage and ΔRg-PIV remained statistically significant in the multivariate model. Table 3 provides a detailed summary of patient distributions and the corresponding analysis results.

### 3.4. Clinical Response and Survival Outcomes

Based on MRI-assessed treatment response, patients were classified into two groups: 32 patients in the C-NCRG (43.8%; mrTRG1:5.5%, mrTRG2:38.4%) and 41 patients in the RDG (56.2%; mrTRG3:52.1%, mrTRG4:4.1%). In the C-NCRG following TNT, mesorectal lymph node involvement was present in 8.2% of patients, pelvic lymph node involvement in 6.8%, EMVI positivity in 11%, and TD positivity in 6.8%. Rectoscopy revealed a clinical complete response in 49.3% of patients upon reevaluation.

At the end of TNT, all patients were discussed by a multidisciplinary tumor board, and 49.3% (*N* = 36) of patients were managed with the NOM protocol, while 46.6% (*N* = 34) underwent surgery. Systemic treatment was modified in 4.1% (*N* = 3) of patients, owing to disease progression with distant metastasis.

In the NOM group, four patients had mrTRG1, 23 had mrTRG2, and nine had mrTRG3 scores. The nine patients reported as mrTRG3 were included in the follow-up protocol because their rectoscopy results were consistent with a complete response. Tumor regrowth was observed in 25% (*N* = 9) of patients managed with the NOM protocol. The median time to regrowth was 164 days (range: 79–491 days). No significant association was observed between regrowth and mrTRG scores.

Among the patients who underwent surgery, five had an mrTRG2 score, 27 had an mrTRG3 score, and four had an mrTRG4 score. Although reported as mrTRG2 on MRI, surgery was performed in five patients who declined the NOM protocol. Pathological results showed ypT0N0 in two patients, while residual disease was pathologically confirmed in the remaining three patients (ypT2N0 in two patients and ypT3N0 in one patient).

**Figure 3:**
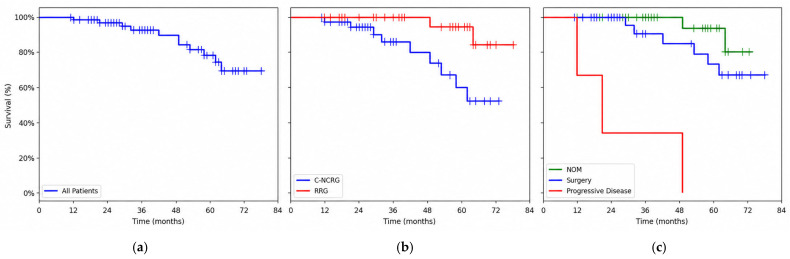
Kaplan–Meier estimates of overall survival: (**a**) all patients, (**b**) MRG response group, and (**c**) management after total neoadjuvant treatment.

In the entire cohort, the 2-year OS was 94.9%, and the 5-year OS was 69.5% (Figure 3a). In the C-NCRG, the 2- and 5-year OS rates were 100% and 84.2%, respectively, and in the RDG, they were 94.5% and 59.6%, respectively, with significantly better outcomes in the C-NCRG (*p* = 0.01) (Figure 3b). There was no significant difference in overall survival between patients who underwent surgery and those who underwent the NOM protocol (*p* = 0.17). In the NOM protocol group, the 2- and 5-year OS rates were 100% and 93.8%, respectively, whereas in the surgical group, they were 100% and 73.1%, respectively (Figure 3c). The median survival of the three patients with progression was 15 months (range, 6–42 months).

## 4. Discussion

In the present study, we demonstrated that lower last-PIVs and a greater reduction in the PIV during treatment (Δ-PIV, defined as last-PIV minus first-PIV) were significantly associated with complete or near-complete MRI response following total neoadjuvant therapy (TNT) in patients with locally advanced rectal cancer. Importantly, within the non-operative management (NOM) cohort, patients without regrowth exhibited a significantly greater decline in the PIV (ΔRg-PIV) compared to those who developed regrowth, suggesting that inflammatory dynamics may also reflect the durability of response under organ-preservation strategies. Together, these findings indicate that treatment-related modulation of systemic inflammation parallels radiological tumor regression and may provide complementary information in both response assessment and longitudinal monitoring in the TNT-NOM era. The observed differences in sensitivity and specificity between the ROC analyses may be explained by differences in endpoint definition and cohort characteristics. The MRI response analysis is based on a more homogeneous imaging-defined outcome, whereas regrowth in the NOM cohort represents a more complex clinical endpoint influenced by multiple factors and a limited number of events. Additionally, the relatively small number of regrowth events may have contributed to variability in the performance metrics observed in the NOM cohort. To our knowledge, few studies have specifically investigated dynamic PIV changes in relation to MRI-based response and regrowth outcomes, supporting the exploratory relevance of our findings.

Each parameter of the PIV reflects the host’s inflammatory status and is also involved in cancer pathogenesis and the metastatic process. The tumor-infiltrating lymphocyte (TIL) score is a well-known biomarker for predicting tumor response and survival in patients with rectal cancer receiving neoadjuvant therapy [22]. As lymphocytes are among the most critical immune cells in the defense against cancer, decreased levels in peripheral blood and lower TIL counts are associated with poor prognostic outcomes [23]. Neutrophils are the first immune cells to be recruited to the site of inflammation. Neutrophils promote carcinogenesis by inducing the release of reactive oxygen species (ROS), leading to DNA damage [24]. In addition, neutrophils produce several mediators, including epidermal growth factor, hepatocyte growth factor (HGF), and platelet-derived growth factor, which can promote tumor growth [25]. Monocytes and their tissue derivatives, including myeloid-derived suppressor cells (MDSCs) and tumor-associated macrophages (TAMs), are commonly present in the tumor microenvironment (TME), where they contribute to tumor progression, angiogenesis, metastatic dissemination, chemoresistance, and immune suppression [26]. The circulating monocyte level may reflect the increased production of tissue macrophages and increased circulating monocytes, which independently predicted incident cancer and mortality [27]. Platelets play important roles in many physiological processes, including inflammation, wound healing, angiogenesis, immune responses, and cancer. Also, platelets are related to cancer dissemination and worse prognosis. There are different biological mechanisms and pathways involving platelets that facilitate tumor metastasis, such as tumor-cell-induced platelet activation and aggregation, promoting tumor evasion of immune destruction, interactions with neutrophils of the tumor microenvironment, inducing an invasive epithelial–mesenchymal transition phenotype of tumor cells, facilitating tumor anoikis resistance and extravasation, and inducing tumor angiogenesis and vascular remodeling [28].

These cells significantly influence cancer development and progression; therefore, inflammatory indices have been investigated as markers associated with treatment response and oncological outcomes. Our PIV formula provides a systemic inflammation point of view, including the ratio of platelets, monocytes, and neutrophils to lymphocytes, and many breakdowns of this formula have been studied separately, mostly based on pretreatment values. The potential use of an increased neutrophil-to-lymphocyte ratio (NLR) as a poor prognostic factor in colorectal cancer was first suggested by Satomi et al. in 1995 [29]. Over the following three decades, numerous studies have demonstrated that an elevated NLR at the beginning of treatment predicts poor overall survival and disease-free survival in patients diagnosed with locally advanced rectal cancer [30,31]. A meta-analysis of 31 studies conducted by Colloca et al. reported that an NLR > 3 is a poor prognostic factor, particularly in elderly rectal cancer patients receiving chemoradiation followed by surgery [32]. Recently, Cui et al. reported that a high pretreatment neutrophil-to-lymphocyte ratio (NLR) (cutoff value > 2.8) was associated with worse OS and DFS in patients with locally advanced rectal cancer who underwent neoadjuvant chemoradiotherapy followed by surgery [33]. The 5-year OS rates were 96.6% and 89.3% in the low-and high-NLR groups, respectively (*p* = 0.016). The 5-year DFS rates in the low- and high-NLR groups were 95.0% and 83.7%, respectively (*p* = 0.029). Furthermore, Hamid et al. indicated that the pretreatment NLR (cutoff value ≥ 4) was associated with worse OS (HR 1.92, 95% CI 1.60–2.30, *p* < 0.001), DFS (HR 1.83, 95% CI 1.51–2.22, *p* < 0.001), and a lower rate of pathologic complete response in patients treated with surgery alone or preoperative CRT followed by surgery [34]. Other parameters with prognostic significance have been investigated as markers of systemic inflammation, including the platelet-to-lymphocyte ratio (PLR) and lymphocyte-to-monocyte ratio (LMR). A systematic review and meta-analysis, which was conducted to evaluate the predictive role of the PLR in the prognosis of patients with rectal cancer undergoing surgery, has demonstrated that a high pretreatment PLR is associated with poorer OS (*p* = 0.01) in patients with rectal cancer undergoing curative resection but not with DFS (*p* = 0.09) [35]. Another systematic review and meta-analysis that included 42 retrospective and five prospective studies revealed that worse overall survival was associated with a high NLR (*p* < 0.001), high PLR (*p* = 0.009), and low LMR (*p* = 0.01); however, worse DFS was found to be associated only with a high NLR and low MLR, not a high PLR [31]. A large retrospective multicentric Italian study, which aimed to evaluate the prognostic and predictive role of several baseline inflammatory markers in LARC patients treated with neoadjuvant CRT, reported significant outcomes [15]. This study evaluated the following parameters: HEI (hemo-eosinophils inflammation index), SII (systemic index of inflammation), NLR (neutrophil-to-lymphocyte ratio), PLR (platelet-to-lymphocyte ratio) and MLR (monocyte-to-lymphocyte ratio). Multivariate analysis indicated that a higher NLR (cutoff value > 1.2) and SII (cutoff value > 500) were related to lower pCR (*p* = 0.05 and 0.009, respectively). In addition, Wang et al. reported a significant correlation between pretreatment biomarkers and pathological responses to neoadjuvant CRT [36]. Multivariate analysis in this study demonstrated that both the PLR and the prognostic nutritional index (PNI) were significant predictors of pathological response. The high PNI group (cutoff value ≥ 45) was significantly higher than the low PNI group (*p* = 0.029). Furthermore, the correlation between the PLR and pathological response was also demonstrated in a prospective study conducted by Manoochehry et al. [37]. An elevated pretreatment PIV (>454.7), combining platelets, monocytes, neutrophils, and lymphocytes, has also been reported to be associated with unfavorable outcomes in rectal cancer [14]. The low pretreatment PIV, NLR, PLR, and MLR, and high LMR in these studies were correlated with better pathological response and outcome, while no comparison was made between the pretreatment and post-treatment values, and whether there was a change in initial levels after treatment. The striking finding of our cohort with the PIV formula, including the NLR, PLR, MLR, and LMR, is directly related to how the PIV changed with treatment and how it was correlated with clinical MR response based on this change. We need to point out that the similar pretreatment PIVs between the CRG and RDG changed significantly after treatment in significant correlation with the objective MR response. Additionally, the observation of lower PIV levels in non-regrowth patients reinforces the hypothesis that these cellular mechanisms contribute to tumor progression and regrowth dynamics.

Dynamic indicators of tumor burden have also been assessed, including circulating tumor DNA (ctDNA). ctDNA in plasma provides a real-time indicator of tumor burden as an established prognostic parameter with clearance of baseline levels during CRT [38], and with tumor progression earlier than radiological imaging for predicting prognosis in LARC [39]. Although it has been demonstrated that patients with undetectable ctDNA after neoadjuvant therapy tend to have better surgical outcomes and are good responders, its role in predicting pathological complete response (pCR) for the NOM protocol remains uncertain [40,41,42]. While ctDNA offers tumor-specific molecular information, the PIV reflects the host’s systemic immune–inflammatory response. Due to the retrospective nature of our trial, we could not correlate dynamic ctDNA and dynamic PIVs with radiological response in our cohort.

Although the mrTRG is a well-established imaging-based marker of treatment response, it may not fully capture the biological mechanisms underlying tumor regrowth, particularly in the context of non-operative management. Therefore, the lack of association observed in this study should be interpreted with caution.

Although overall survival differences were observed between MRI response groups, this study was not designed to evaluate the independent prognostic value of PIV dynamics for survival outcomes. Therefore, no conclusions regarding survival prediction based on PIV dynamics should be drawn from the present analysis.

This study had several limitations. Our retrospective design may have introduced selection bias and restricted our ability to establish causality. Given the relatively small sample size and limited number of events, particularly within subgroup analyses, the present findings should be interpreted with caution. The risk of overfitting in multivariable models cannot be excluded, and the observed associations should be considered exploratory and hypothesis-generating. External validation in larger, independent cohorts is required before these findings can be translated into clinical practice. In addition, the cutoff values for the Δ-PIV and ΔRg-PIV were derived using ROC analysis within the same cohort and may therefore be subject to optimistic bias. These thresholds should be considered exploratory and cohort-specific and require validation in independent datasets. Investigation of the optimal timing of PIV measurement during TNT to maximize its predictive value may highlight the potential for personalized medicine approaches, where PIV dynamics could help tailor TNT protocols or guide decisions on non-operative management versus surgery for individual patients. The individual contribution of each PIV component (platelets, monocytes, neutrophils, and lymphocytes) was not separately analyzed in this study, which may limit the understanding of the underlying biological mechanisms. Future studies are needed to better elucidate the specific role of each component in treatment response. Although the Δ-PIV demonstrated a statistically significant association with MRI response, its moderate discriminative performance and relatively low sensitivity limit its use as a standalone predictive biomarker. Instead, it may serve as a complementary tool alongside established clinical and imaging modalities in the assessment of treatment response. Furthermore, comparative studies integrating dynamic PIVs with other emerging biomarkers, such as circulating tumor DNA (ctDNA), and advanced imaging techniques, such as radiomics and AI, are crucial for developing a comprehensive multimodal assessment strategy for LARC response. Heterogeneity in total neoadjuvant therapy regimens may influence systemic inflammatory responses and act as a potential confounding factor. Given the limited sample size, stratified analyses were not feasible, and this variability should be considered when interpreting the findings. As patients with mucinous tumors were excluded from the analysis, our findings may not apply to this subgroup of patients. For patients without regrowth, the use of a surrogate timepoint based on the cohort’s median time to regrowth may introduce bias and limit comparability between groups. Therefore, findings related to the ΔRg-PIV should be interpreted cautiously and considered exploratory. Detailed pathological features such as tumor differentiation and lymphovascular invasion were not consistently available due to the limitations of pretreatment biopsy specimens and therefore could not be included in the analysis. Further stratification of nodal status (e.g., N1 vs. N2) was not feasible due to the limited sample size and subgroup distribution, so should be explored in future larger studies.

## 5. Conclusions

This study suggests that dynamic changes in the PIV may represent a potential exploratory and complementary biomarker reflecting the host’s systemic immune response. However, given the moderate predictive performance, PIV dynamics should not be considered a standalone tool and require validation in larger independent cohorts before clinical application.

## Figures and Tables

**Table 1:** Criteria for response assessment on post-neoadjuvant chemoradiotherapy.

MR-TRG	T2-HR-MRI	DWI	Combination of T2-HR and DWI
**1**	Normal rectal wall or thin band of fibrosis	No foci of restricted diffusion	T2-HR 1/2/3 + DWI 1
**2**	Thick band of fibrosis with doubtful residual tumor	Few scattered foci of restricted diffusion	T2-HR 2/3 + DWI 2
**3**	Fibrosis/mucin > 50% with tumor	C-shaped band or nodular focus of restricted diffusion	T2-HR 2/3 + DWI 3
**4**	Little fibrosis, mostly tumor	Smaller than pre-Rx MR	T2-HR 4 + DWI 4
**5**	No response or progression	No change since previous	T2-HR 5 + DWI 5

Abbreviations: MR-TRG: Magnetic Resonance–Tumor Response Group; T2-HR: T2-high resolution; DWI: diffusion-weighted imaging.

**Table 2:** Patients’ characteristics and univariate/multivariate analysis outcomes in the cohort.

Characteristics	All Patients (*N* = 73)	C-NCRG (*N* = 32)	RDG (*N* = 41)	Univariate *p*-Value	Multivariate *p*-Value	OR (95% CI)
Age, median (range)	59 (36–82)	60 (36–77)	59 (36–82)	-	-	
Gender						
Female	29 (39.7%)	10	19	-	-	
Male	44 (60.3%)	22	22			
Tumor Location						
Distal	26 (35.6%)	13	13			
Middle/Proximal	47 (64.4%)	19	28	0.431	-	-
Initial MRI						
T stage						0.512
T3a−b	42 (57.5%)	24	18	0.009	0.306	(0.142–1.846)
T3c−d/T4a−b	31 (42.5%)	8	23			
Initial MRI Mesorectal Node						
No	26 (35.6%)	16	10	0.026	0.262	0.502
Yes	47 (64.4%)	16	31			(0.151–1.672)
Initial MRI Pelvic Node						
No	67 (91.8%)	31	36	0.193	0.852	0.766
Yes	6 (8.2%)	1	5			(0.047–12.491)
Initial MRI EMVI						
No	46 (63%)	24	22	0.064	0.634	0.703
Yes	27 (37%)	8	19			(0.165–2.996)
Initial MRI Tumor Deposits						
No	61 (83.6%)	29	32	0.161	0.437	0.426
Yes	12 (16.4%)	3	9			(0.049–3.664)
Δ-PIV, mean ± SD	−59.74	−197.2	47.49	0.006	0.007	0.997
	± 352.27	± 210.7	± 402.5			(0.994–0.999)

Abbreviations: C-NCRG: complete/near complete response group; RDG: residual disease group; N: Number of patients; MRI: magnetic resonance imaging; EMVI: extramural venous invasion, OR: odds ratio; PIV: Pan-Immune-Inflammation Value; SD: standard deviation.

**Table 3:** Univariate and multivariate analysis outcomes in the NOM cohort.

Characteristics	All NOM (*N* = 36)	Regrowth (*N* = 9)	Non-Regrowth (*N* = 27)	Univariate *p*-Value	Multivariate *p*-Value	OR (95% CI)
Tumor Location Distal Middle	16 (44.4%) 20 (55.6%)	2 7	14 13	0.13	0.152	7.463 (0.476–116.883)
Initial MRI T Stage T3a-b T3c-d/T4a-b	28 (77.8%) 8 (22.2%)	4 5	24 3	0.01	0.008	64.958 (2.996–1408.340)
Initial MRI Mesorectal Node No Yes	17 (47.2%) 19 (52.8%)	2 7	15 12	0.97	-	-
Initial MRI Pelvic Node No Yes	34 (94.4%) 2 (5.6%)	8 1	26 1	0.423	-	-
Initial MRI EMVI No Yes	27 (75%) 9 (25%)	6 3	21 6	0.50	-	-
Initial MRI Tumor Deposits No Yes	33 (91.7%) 3 (8.3%)	8 1	25 2	0.72	-	-
ΔRg-PIV, mean ± SD	−34.9 ± 260.73	169.71 ± 327.76	−103.23 ± 197.79	0.02	0.014	1.009 (1.002–1.016)

Abbreviations: NOM: non-operative management; N: Number of patients; MRI: magnetic resonance imaging; EMVI: extramural venous invasion; Rg-PIV: Regrowth Pan-Immune-Inflammation Value; SD: standard deviation.

## Data Availability

The data supporting the findings of this study are available from the corresponding author upon reasonable request. The data are not publicly available due to privacy and ethical restrictions involving patient information.

## Abbreviations

AUC	Area Under the Curve
CRT	Chemoradiotherapy
ctDNA	Circulating Tumor DNA
DFS	Disease-Free Survival
EMVI	Extramural Venous Invasion
LARC	Locally Advanced Rectal Cancer
MRI	Magnetic Resonance Imaging
mrTRG	Magnetic Resonance Tumor Regression Grade
NOM	Non-Operative Management
OS	Overall Survival
pCR	Pathological Complete Response
PIV	Pan-Immune Inflammation Value
ROC	Receiver Operating Characteristic
TME	Total Mesorectal Excision
TNT	Total Neoadjuvant Therapy
